# Taxonomic identity best explains variation in body nutrient stoichiometry in a diverse marine animal community

**DOI:** 10.1038/s41598-020-67881-y

**Published:** 2020-08-13

**Authors:** Jacob E. Allgeier, Seth Wenger, Craig A. Layman

**Affiliations:** 1 Department of Ecology, and Evolutionary Biology, University of Michigan, Ann Arbor, MI USA; 2 Odum School of Ecology, University of Georgia, Athens, GA USA; 3 Department of Applied Ecology, North Carolina State University, Raleigh, NC USA

**Keywords:** Ecology, Ecology

## Abstract

Animal-mediated nutrient dynamics are critical processes in ecosystems. Previous research has found animal-mediated nutrient supply (excretion) to be highly predictable based on allometric scaling, but similar efforts to find universal predictive relationships for an organism’s body nutrient content have been inconclusive. We use a large dataset from a diverse tropical marine community to test three frameworks for predicting body nutrient content. We show that body nutrient content does not follow allometric scaling laws and that it is not well explained by trophic status. Instead, we find strong support for taxonomic identity (particularly at the family level) as a predictor of body nutrient content, indicating that evolutionary history plays a crucial role in determining an organism’s composition. We further find that nutrients are “stoichiometrically linked” (e.g., %C predicts %N), but that the direction of these relationships does not always conform to expectations, especially for invertebrates. Our findings demonstrate that taxonomic identity, not trophic status or body size, is the best baseline from which to predict organismal body nutrient content.

## Introduction

Animals are important drivers of nutrient dynamics in many ecosystems^1–4^. Nutrients frequently limit key ecosystem processes, such as primary production^5^, and animals can represent a major source of labile nutrients through their excretion, at times alleviating nutrient limitation^6–8^. Because they are often the dominant pool of biomass (particularly in many aquatic systems), animals can also represent an important reservoir of nutrients stored in their tissue^9^. While previous work has shown the supply of nutrients from animals via excretion is highly predictable based on rules of allometry^10–12^, no universal factor has been found to broadly predict the amount of nutrients stored in the body tissue of organisms^13–18^.

Animal body nutrient composition, an indicator of an organism’s relative demand for nutrients, is a product of myriad evolutionary, ecological, and environmental factors^19,20^. Structural characteristics, such as phosphorus (P)-rich bones or nitrogen (N)-rich carapaces or spines, are often phylogenetically conserved and can contribute substantially to the nutritional makeup and demands of an animal. Ecological (e.g., diet) and environmental (e.g., temperature) factors, on the other hand, influence physiological processes and may also affect body nutrient composition^21,22^. The high degree of interdependence of ecological and evolutionary influences makes identifying specific factors that drive patterns of nutrient content in animals difficult. For example, the Growth Rate Hypothesis (GRH) proposes that body P content is highly predicted by growth rate because a higher growth rate requires higher levels of P-rich RNA^23,24^. However, this hypothesis was developed from studies of organisms with low structural demand for P, e.g., invertebrates^23^, and is less supported in vertebrates. This interaction between evolutionary and ecological processes limits the predictability of body nutrient content^15,17,18,25–27^, but suggests that such processes are ultimately contingent on the taxonomic identity of organisms^28,29^.

Researchers have sought to identify predictable relationships of body nutrient content across taxa using three primary frameworks. First, allometric relationships are generalizable across highly diverse taxa^30^, and for a wide array of ecological processes, based on predictable fractal scaling associated with metabolic rates—the Metabolic Theory of Ecology (or MTE)^31^. However, it is less clear that such relationships apply to the *storage* of nutrients in somatic tissues. Empirical support for body size-body nutrient content relationships has been mixed^18,27,32–34^. Davis and Boyd^32^ and Gonzalez et al.^18^ found positive, negative, and non-significant relationships between single elements and body size in fish and invertebrates. Sterner and George^35^ and El-Sabaawi et al.^26^ found mixed (negative and non-significant) support for body nutrient content and body size relationships in fish. Others, including Fagan et al.^14^ and Lemoine et al.^17^, found mixed and weak support across groups of invertebrates.

Second, the trophic status of organisms has been identified as a useful predictor of body nutrient content in arthropods and other invertebrates^18,25^, temperate and tropical fishes^15^, and across other taxa^17^. This suggests that organisms, over evolutionary time, reduced the stoichiometric imbalances between their body tissues and their prey to maximize utilization of their food resources^36^. This concept is a basic tenet of Ecological Stoichiometry Theory (EST) and suggests that animals within trophic groups are inherently limited by the quality of their food resource (plant matter is rarely as nutrient-rich as animal tissue)^19^, and body nutrient composition has indeed been shown to largely reflect diets^16,26,37^. For example, herbivores and detritivores feed on resources with high C:nutrient ratios, and also tend to have higher body C:nutrient ratios than organisms at higher trophic levels^15,18,38^. This suggests an inverse relationship between trophic position and body C:nutrient ratios.

A third avenue for predicting organismal body nutrient content is using one body nutrient to predict another, i.e., examining how nutrients covary. Hendrixson et al.^15^ found relationships between body content of C and P were negatively correlated across multiple species of temperate fishes. This approach has not received widespread attention (but see^18^), but does offer important insight into the degree to which elements are fundamentally linked, whereby the demand for one nutrient coincides with the demand for another^15,38–40^, herein termed “stoichiometrically linked.” One expectation would be a negative relationship between elements, as found by Hendrixson et al.^15^, as this suggests net conservation of elemental mass-balance, whereby increases in one nutrient results in reductions in another. Further, if trophic group is a good predictor of body stoichiometry then the relationship (i.e., the slope) by which elements are stoichiometrically linked may differ across trophic groups or other levels of classification that have large differences in body nutrient content. One example of this is the difference between vertebrates and invertebrates, whereby the P-rich internal skeleton of vertebrates should lower body C:P and N:P ratios, and thus generate relationships between these pairs of elements with steeper slopes.

We take advantage of a large dataset of somatic carbon (C), nitrogen (N), phosphorus (P), and their ratios for 738 individuals of 105 invertebrate and vertebrate species (52 families, 68 genera) in a single tropical marine community to test for predictive relationships in body nutrient content. We ask three questions:

- *Question 1: Does the trophic level of an organism explain variation in body nutrient content, such that body C:nutrient ratios decrease with increasing trophic position? We also test an alternative hypothesis that taxonomic identity is a superior predictor of body nutrient content than trophic level.*
- *Question 2: Does body mass improve prediction of body nutrient content beyond trophic level or taxonomic identity alone? We test the hypothesis that body nutrient content scales allometrically.*
- *Question 3: Are body nutrient concentrations (C, N, P) stoichiometrically linked? We test the hypothesis that body C is negatively related to N and P. We further hypothesize that the slopes of these relationships will vary predictably according to trophic level classification.*

A key strength of our analysis is that all organisms come from the same ecological community, eliminating any confounding effects of variation in environmental factors such as temperature and resource availability. This provides an alternative and complementary approach to meta-analyses or other data compilations across ecosystems and study organisms.

## Methods

**Figure 1:**
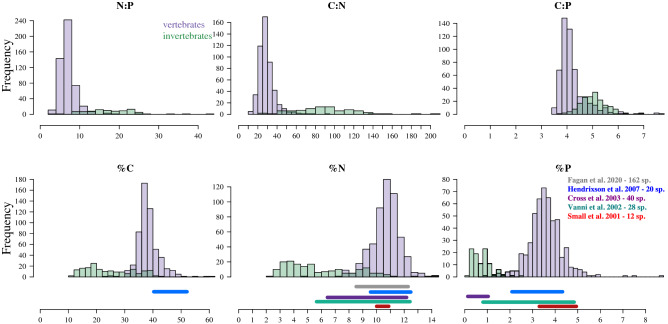
Histograms of percent nutrient content for carbon (C), nitrogen (N), and phosphorus (P), and their ratios for invertebrates (light green) and vertebrates (purple). The bars below indicate the range of values reported in the studies of the corresponding color. For each study we indicate the number of species used in the analysis. The current study analyzed 105 species.

Individual organisms were collected using hook and line, traps, cast nets, and dip nets between 2008 and 2011, within the same large ~ 12 km^2^ embayment (the Bight of Old Robinson) on Abaco Island, The Bahamas (Allgeier et al.^10^). Habitat types within the embayment consisted of seagrass, mangrove, and coral complexes. This system is a continuum of habitats without clear boundaries, partly because there are no rivers and thus no brackish estuaries; as a result, patches of coral can be found within mangrove and seagrass ecosystems. Nearly all, if not all, the organisms collected in the embayment could be found within any one of these sub-ecosystem types. Of the 738 individuals (51 families, 68 genera, 105 species), 195 were invertebrate and 543 were vertebrates. Invertebrate species collected were diverse in taxonomy (22 families, 26 genera, 31 species) and morphology/physiology—including shrimp, jellyfish, and gastropods, among many other groups. Vertebrates were also very diverse (30 families, 42 genera, 74 species), and included organisms with rather different morphologies, such as moray eels (e.g., *Gymnothorax funebris*), and pipefish (e.g., *Cosmocampus brachycephalus*). For comparison with published body nutrient content data, we extracted the range of values for %C, %N, and %P from the five studies cited in Fig. 1.

Individuals were identified, weighed for wet mass, and measured to standard length. Animals were either dissected to remove stomach contents (fish) or allowed to incubate for 6–12 h to clear gut passage (invertebrates) and then frozen for transport to Odum School of Ecology and processed for elemental content (see below). Samples were lyophilized to a consistent dry weight then ground to a powder with a ball mill grinder. Larger individuals required blending to homogeneity before mill grinding. Ground samples were analyzed for %C and %N content and δ^15^N (as a proxy for trophic position; see below) with a CHN Carlo-Erba elemental analyzer (NA1500) CN Analyzer and for %P using dry oxidation-acid hydrolysis extraction followed by colorimetric analysis (Alpkem RF300). Elemental content was calculated on a dry weight basis, with nutrient ratios expressed on a molar basis.

To create a categorical variable for trophic level (herein Trophic Group, TG, used in Questions 1, 2, and 3), each organism was placed into one of eight trophic groups: detritivore, herbivore, microinvertivore, macroinvertivore, piscivores-invertivore, piscivores, zooplanktivore, and omnivore. Classifications were designated based on Newman et al.^41^ and Munro^42^ for fish and invertebrates, and the authors’ data and observations from this system (Allgeier and Layman *unpublished*). δ^15^N values were used as a continuous measure for the trophic level of an individual (Questions 2), following the rationale that the higher the δ^15^N value the higher on the food chain an individual is feeding^43–46^. We did not correct for potential spatio-temporal variation in isotopic baselines for three reasons: (1) the myriad basal resources within these systems makes correcting for specific isotopic baselines exceedingly difficult^43,45^, (2) we are not attempting to use these data to calculate trophic position sensu stricto and instead were interested in relative trophic position among focal taxa, and (3) previous research using the same species from the same study area found clear gradients of δ^15^N values that accurately depict relative trophic levels in this system with relatively little inter-annual variation in isotopic values among consumers^44^. We acknowledge potential error associated with this approach, but suggest the continuous nature of δ^15^N renders it preferable to simply assigning species to exact trophic levels based on presumed natural history or literature sources from studies conducted in other systems. The University of Georgia’s Institutional Animal Care and Use Committee approved protocols for the capture and handling of fish (AUP # A2009-10003-0). All methods were carried out following relevant guidelines and regulations.

### Statistical methods

*Question 1*: We used simple linear regression to test the degree to which every nutrient and nutrient ratio was explained by an individual’s trophic group. Because previous work has indicated the importance of taxonomy for predicting animal-mediated nutrient dynamics, we also tested the relative importance of each of the following predictors: vertebrate/invertebrate, class, order, family, genus, and species (e.g., *% C* ~ *family*). We, therefore, tested six competing models for each of the six response variables (C, N, P, N:P, C:N, and C:P). Comparisons among competing models were made using the Akaike information criterion adjusted for small sample size (AICc) and the *r*^2^ statistic^47,48^. Sample sizes were always the same; i.e., any sample that had a %N value had corresponding data for all levels of taxonomic identity. All assumptions for general linear models were met for these analyses. These and subsequent analyses were conducted using R software^49^.

*Question 2*: To test for the importance of body size on body nutrient content and stoichiometry, we ran mixed-effects models using mass as a fixed effect with a random slope of taxonomy following^10^. For taxonomy, we used the best supported trophic group or taxonomic level from our statistical test for *Question 1*. Statistical tests for *Question 1* and *Question 2* were conducted separately rather than simultaneously with a global model (e.g.,^10^) because our tests of such global models showed frequent failure to converge. We also tested the importance of trophic group (TG) and δ^15^N (as a proxy for trophic position) by adding all combinations of these terms and mass as fixed effects to models with a random effect of taxonomy (again based on the level determined from *Question 1*). Comparisons among competing models were made using AICc and the *r*^2^ statistic (*r*^2^_total_; which includes fixed and random effects, as well as *r*^2^_fixed_ for fixed effects alone^50^). Mixed-effects models were run using the lme4 package in R^51^.

*Question 3*: We used linear mixed-effects models to conduct three tests regarding the extent to which single elements were stoichiometrically linked. First, we tested for a significant relationship between elements (i.e., N ~ P, C ~ N, C ~ P). Second, to test if these relationships differed among vertebrates and invertebrates, an interaction term was included in the model (N or P × vertebrate/invertebrate). Finally, to test if these relationships were best explained at the family or trophic group level, we ran two separate models with the same fixed effects (body N or P, vertebrate/invertebrate, and their interaction), but with different random effects, either for family-level taxonomy (determined from *Question 1*) or for trophic group. For each class of model (with random effects for family or trophic group) we identified the best model using AICc. The best family-level or trophic group-level models were then compared using AICc. We also calculated Pearson’s correlation coefficients for the relationships between elements (C:N, C:P, N:P) for invertebrates, vertebrates, and both groups combined. All assumptions were met for mixed-effects models.

## Results

We used a total of 738 individuals from 105 species, 68 genera, 51 families, and 8 functional feeding groups in our analyses. The vertebrate assemblage collected for this analysis accounted for ~ 46% of all species observed across 122 surveys of coral reef, seagrass, and mangrove locations in the Northern Antilles, but represented > 90% of the biomass among these communities^52^. On average, there were 2 species per family (range 7–1 species; 26 families had one species). On average, there were 14 individuals per family, fifteen families (29%) had fewer than three individuals, and six (11%) had one individual.

Variation in body nutrient content ranged substantially (%N: 2.32–14.22, %P: 0.19–8.7, %C: 11–60.1, NP: 2.9–128.2, C:N: 3.5–7.7, C:P: 11.3–562.2) relative to previous community-level studies: Small et al.^53^: %N ~ 10.1–10.6, %P ~ 3.6–5, 12 species, 4 families; Cross et al.^21^: %N ~ 6–12, %P 0–1.8, 40 species; Vanni et al.^54^: %N ~ 6.5–12, %P ~ 0.75–5, 28 species, 13 families; Hendrixson et al.^15^: %N ~ 9.3–12.2, %P ~ 2–4.1, %C ~ 40–52, 20 species and meta-analyses; Fagan et al.^25^: %N ~ 8–12, 162 species, 65 families (Fig. 1).

**Figure 2:**
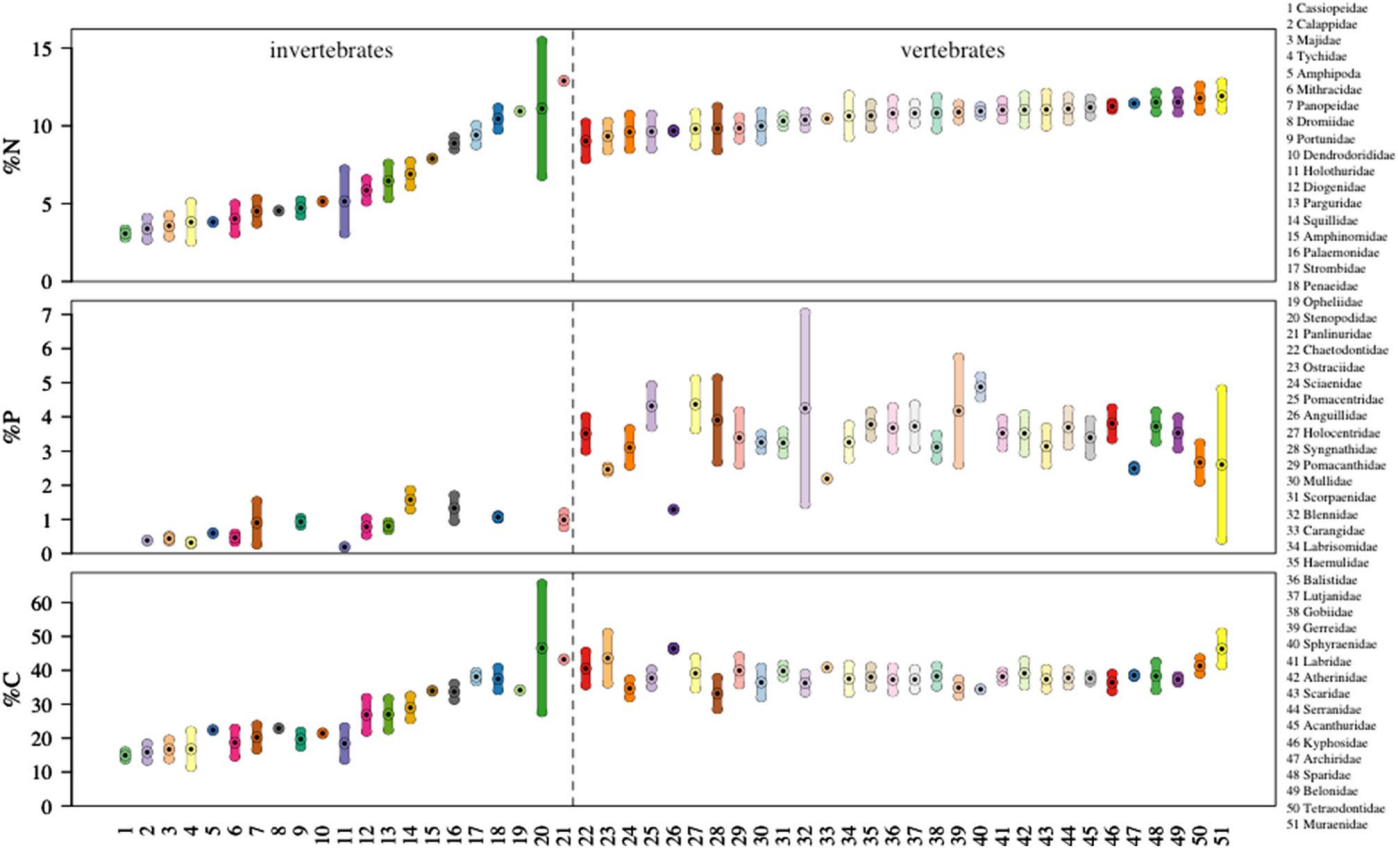
Raw values for C, N, and P across families delineated by invertebrates and vertebrates. Bars indicate standard deviation around the mean (center point with black dot). Family names are associated with numbers on the x-axis and the list on the right side of the figure (having a consistent color in all three plots). Families are ordered from smallest to largest %N body content for all three plots for ease of comparison across plots.

*Question 1*: Differences in body nutrient content (%N, %P, %C) were best explained at the level of family, as was body nutrient stoichiometry for C:P, based on lowest AICc (Fig. 2 and Table 1). Differences in body nutrient stoichiometry for N:P and C:N were best explained at the genus level (Fig. 2). In each case, the best model explained a large proportion of the variation in the data: > 80% for all response variables, except for C:P for which 78% of the variation in the data was explained.

**Table 1:** Model comparisons of the relative ability of taxonomic levels (species, genus, family, and class), invertebrate/vertebrate category, and trophic groups to explain diffence in body nutrient content of organisms.

Tax.level	%N		%P		%C		N:P		C:P		C:N	
	*r*^2^	AICc	*r*^2^	AICc	*r*^2^	AICc	*r*^2^	AICc	*r*^2^	AICc	*r*^2^	AICc
Vert/invert	0.57	− 407	0.73	− 28.73	0.49	− 519.25	0.06	− 490.36	0.00	− 741.30	0.16	90.33
Class	0.67	− 608.82	0.92	− 775.34	0.64	− 788.79	0.06	− 483.25	0.13	− 838.98	0.16	99.77
Order	0.85	− 1,185.76	0.94	− 917.17	0.81	− 1,247.84	0.10	− 493.76	0.72	− 1667.27	0.23	59.79
Family	**0.89**	**− 1,377.44**	**0.96**	**− 1,240.00**	**0.85**	**− 1,407.79**	0.79	− 1,316.31	**0.78**	**− 1,820.06**	0.79	− 648.96
Genus	0.87	− 1,229.57	0.94	− 919.33	0.82	− 1,240.62	**0.92**	**− 1886.89**	0.71	− 1572.02	**0.83**	**− 768.50**
Species	0.80	− 866.61	0.89	− 490.67	0.78	− 1,050.85	0.80	− 1,272.10	0.63	− 1,351.83	0.78	− 575.51
Trophic group	0.25	20.42	0.25	617.83	0.20	− 177.35	0.09	− 498.91	0.09	− 803.18	0.12	122.74

Bold text indicates models with the best *r*^*2*^ and AICc.

**Figure 3:**
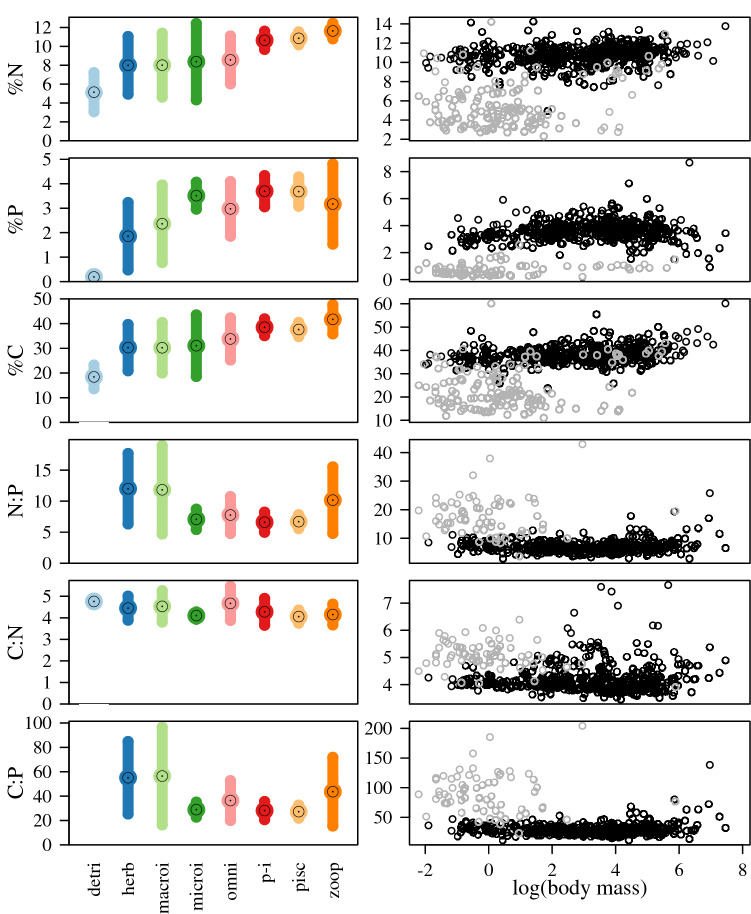
Left panels: Body nutrient content and stoichiometry across trophic groups. Bars indicate standard deviation around the mean (center point with black dot): “detri” = detritivore; “herb” = herbivore; “macroi” = macroinvertivore; “microi” = microinvertivore; “omni” = omnivore; “p-i” = piscivore and invertivore; “pisc” = piscivore; “zoop” = zooplanktivore. Right panels: data for log(body mass) and body nutrient content and stoichiometry. Note mass data was log10 transformed in analysis. Colors indicate datasets: gray = invertebrates only, black = vertebrates only.

*Question 2*: Body mass was a weak predictor of body %N, %P, and N:P, but was retained in the top four models for %C, C:N, and C:P (Table 2; Fig. 3). In all cases, this parameter explained very little of the variance in the data. Only in the cases of %C and C:N did models with body mass have AICc values more than two points better than models without it (Table 2). For all response variables, the inclusion of other covariates had only a marginal effect on model performance relative to the random effects of taxonomy, which explained between 66% (C:N) and 95% (%P) of the variance in the data. Trophic group generally explained the most variance of all fixed effects, and appeared to be the most effective in the models for %P, %N, and N:P, but only in the case of %N was it retained in all models with ΔAIC < 2. The parameter δ^15^N had essentially no effect on model performance, e.g., it was not retained in all models with ΔAIC < 2 for N:P and C:P, but was the only model with ΔAIC < 2 for %C (Table 2).

**Table 2:** Statistics for mixed-effects model output and rank for body nutrient composition that include random effects of taxonomic level and ecological covariates: Mass = wet body mass (continuous; log10 transformed), δ^15^N (continuous), and TG = trophic group classifications (categorical).

Response	Tax. level	Model rank	Mass	δ^15^N	TG	*r*^*2*^_*fixed*_	*r*^*2*^_*total*_	LogLik	AICc	AAIC	Weights
N	Family	1	–	–	1	0.14	0.92	200.01	− 379.68	0	0.38
		5	–	–	–	–	0.92	190.21	− 374.38	5.31	0.03
P	Family	1	–	–	1	0.21	0.95	205.64	− 390.87	0	0.26
		5	–	–	–	–	0.95	197.54	− 389.03	1.84	0.10
C	Family	1	0.12 (0.04)	0.11 (0.04)	–	0.04	0.87	79.14	− 148.19	0	0.85
		7	–	–	–	–	0.88	64.43	− 122.83	25.37	0.00
N:P	Genus	1	0.08 (0.05)	− 0.11 (0.05)	1	0.24	0.82	− 67.92	160.44	0	0.37
		7	–	–	–	–	0.85	− 83.64	173.32	12.88	0.00
C:N	Genus	1	0.20 (0.05)	–	–	0.03	0.71	− 229.43	466.91	0	0.56
		5	–	–	–	–	0.66	− 236.04	478.11	11.20	0.00
C:P	Family	1	0.13 (0.05)	− 0.07 (0.05)	–	0.01	0.87	− 45.90	101.91	0	0.35
		5	–	–	–	–	0.87	− 50.64	107.33	5.42	0.02

*r*^*2*^_*fixed*_ and *r*^*2*^_*total*_ indicates the variation in the data explained by the fixed effects and the full model, respectively^50^. For Mass and δ^15^N, values are parameter estimates (with standard error). For TG, a “1” indicates inclusion in the model (estimates not shown).

**Figure 4:**
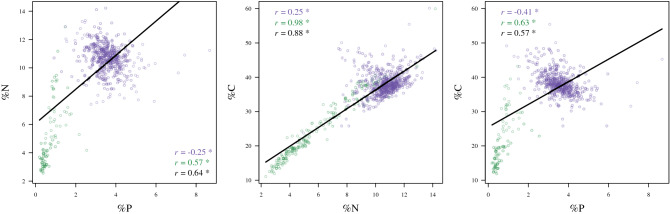
Correlations of stoichiometric relationships between elements. Colors indicate datasets: green = invertebrates only, purple = vertebrates only, black = full dataset. Text in each plot indicates the correlation, * = significant at alpha < 0.05. Dashed black line indicates a slope of 1 for perspective.

*Question 3*: There was a significant relationship between all elements, and in all cases the slope of this relationship was significantly different for vertebrates and invertebrates (i.e., a significant interaction term; Table 3). In all cases, models including the random effect for family outperformed those containing random effects for trophic group; however, in most cases, both of these levels of classification explained a substantial proportion of the variation in the data much more than the fixed effect (Table 3). Correlations between elements were significant when all animals were grouped, as well as when invertebrates and vertebrates were analyzed independently (Fig. 4). The direction of the N:P and C:P relationships differed between invertebrates and vertebrates.

**Table 3:** Statistics for mixed-effects models of stoichiometric relationships between elements.

Relationship	Level of org	# Obs	%Nut	V/I	%Nut * V/I	*r*^*2*^_fixed_	*r*^*2*^_total_	AICc	Weight
N ~ P	Family	444 V/65 I	0.44	3.27	− 0.84	0.69	0.93	− 148.58	1.00
	Trophic	444 V/65 I	1.08	4.55	− 1.61	0.55	0.91	33.62	1.00
C ~ P	Family	434 V/65 I	0.24	0.65	− 0.42	0.63	0.88	− 1,066.31	1.00
	Trophic	434 V/65 I	0.38	0.69	− 0.56	0.71	0.87	− 934.52	1.00

All models include fixed effects for % body nutrients (% Nut; continuous), vertebrate or invertebrate (V/I; categorical), their interaction (%Nut * V/I), and random effects at either family or trophic group level of organization. Lower AICc scores indicate the better model; *r*^*2*^_*fixed*_ and *r*^*2*^_*total*_ indicate the variation in the data explained by the fixed effects and the full model, respectively. The taxonomic level of classfication that provided by the best model is in bold. Mean parameter estimates are shown for %Nut, V/I, and % Nut * V/I.

## Discussion

Developing a predictable understanding of the factors that control how nutrients flow through ecosystems is an important challenge in ecology. We took advantage of an extensive dataset from a diverse marine community of vertebrates and invertebrates in a single ecological system that allows novel insight into the ability to explain the variation in the storage of nutrients by animals in their tissues. We found that animal body nutrient concentration was poorly explained by the basic traits of an individual organism (e.g., body size), but instead was best explained by the taxonomic rank of family. This finding suggests that our ability to predict the storage of nutrients in animals is minimal without a priori knowledge of family-level body nutrient concentrations. However, building on a somewhat neglected approach (and basic tenets of ecological stoichiometry theory), we show that correlations between single elements may provide an alternative avenue of prediction.

Previous studies have found trophic position to be a well-supported predictor of body nutrient content. This idea is rooted in EST, based on the principle that organisms should evolve to minimize the imbalances between their nutritional demands (their body nutrient content) and their diet. In theory, this principle encompasses both ecological (i.e., diet) and evolutionary drivers^36^. Our study confirms previous findings that trophic level can explain some of the variation seen in the real world^17,18,25^. Notably, the most distinct trophic group was detritivores, represented by two individuals of one species, *Holothuria floridana*, a sea cucumber. Interestingly, this species has extremely low %P (~ 0.2%; the lowest in our dataset), %C (15%, 22%), and %N (4%, 7%), numbers that set this species apart as being among of the lowest in all three body nutrients (values below 3%, 34%, 9% represent the 1st quartiles for P, C, and N). Theory would predict that organisms should evolve to minimize the stoichiometric mismatch between body nutrient content and dietary nutrient content. Because detritivores and herbivores consume resources with high C:nutrient ratios, they should also have high C:nutrient ratios, thus spurring the notion *you are what you eat*. Our data show that detritivores do appear to have lower body nutrient content and ratios than predators. However, an important caveat is that there is only one species with two individuals represented in the detritivore group (an invertebrate), whereas there are 22 species (with 158 individuals) of herbivores, which have slightly lower body nutrient content, but still substantially overlap with higher level consumers (Fig. 4).

In contrast, zooplanktivores (including two species, *Atherinomorus stipes*, silversides fish, and *Cassiopea andromeda,* a jellyfish) that feed on invertebrates of low nutrient content (relative to piscivores), had, on average, slightly higher body nutrient content than piscivores. Further, we found that the level of explanatory power of trophic group as a predictor of body nutrient content is minimal compared to differences among families (and/or genus). These findings, in conjunction with others (e.g.,^35^), support the idea that evolutionary processes, such as the development of particular structures to deter predation or those that relate to sexual selection, outweigh ecological factors in determining body nutrient content^55,56^.

We found that family was overall best supported as the taxonomic level at which body nutrient content is conserved, although genus was a better predictor of some nutrient ratios. This finding is consistent with past studies testing EST^10–12^ and inconsistent with others. For example, Gonzalez et al.^18^ found that the level of taxonomy that best predict body nutrient content was dependent on the element in question, and Wiesenborn^57^ found P in invertebrates was best predicted by order. Interestingly, two independent analyses of vertebrates and invertebrates^10,11^ that found the family of the organism (in addition to body size) to be the strongest predictor of excretion rates of N and P, also found that body nutrient content was a poor predictor of nutrient excretion. Taken at face value, this finding is somewhat inconsistent with EST. However, considering our finding that body nutrient content is strongly correlated with family-level taxonomy suggests that the importance of body nutrient content for excretion could have been masked when controlling for the importance of family-level taxonomy in the analysis. Improving the resolution of the excretion analysis by including organismal phylogeny may help clarify past results. Either way, the consistency across these data-rich analyses strongly supports the idea that taxonomy is a key determinant of stoichiometry.

Extending our analysis to include any combination of continuous (body size and δ^15^N) and categorical (trophic group) predictors, while still accounting for variation in taxonomy through the use of random effects, yielded models with only marginal improvements over the basic taxonomic models. These findings are consistent with some studies^32,35^, and in contrast to others^17^, especially when considering the potentially divergent stoichiometry found among conspecific individuals^37^. Although we had some within-species replication in our data, it was not consistent enough to provide a robust test of individual-level variation (e.g., some species had only a single individual, some had > 30). However, the high taxonomic diversity encompassed by our dataset, and the substantial range in body sizes it includes (0.04–2,597 g), lends confidence to our findings: beyond taxonomy, neither body size nor any obvious continuous covariate is a good predictor of body nutrient content. This finding is important for theory and conservation, as it suggests that once data are available for a particular family, body chemistry can be predicted with reasonable confidence for members of that family. However, generating stoichiometry data is time- and cost-intensive and represents a substantial challenge when studying diverse communities, such as those found in the tropics, whereas relationships with continuous variables (e.g., body size) require substantially fewer baseline requirements. Nonetheless, our study represents an important advance by clearly distinguishing the family level of classification as key for predicting body nutrient content.

Despite the lack of predictive power found with continuous covariates, our study showed that elemental composition was generally well predicted by other elements across the diverse community. Previously, Hendrixson et al.^15^ reported strong relationships between %C and %P (negative), and a lack of significance between %C and %N, but the authors were cautionary about their findings in that they were limited to only relatively few temperate fish species. Using a more comprehensive dataset we found significant relationships between all corresponding elements. Because C, N, and P make up a relatively large proportion of the total mass of an organism, a negative relationship between elements could be expected because increasing amounts of one element should come at the expense of another (i.e., if organisms were made up exclusively of C, N, and P, then we would expect perfect relationships among these elements). We found the expected negative relationship for vertebrate C and P and vertebrate N and P, but not vertebrate C and N (Fig. 4). For invertebrates, all relationships were positive rather than negative. Most notable was the tight positive relationship between C and N for invertebrates (*r*^2^ = 0.98).

One hypothesis to explain this result is that many invertebrates have a high proportion of chitin, an N- and C-rich polysaccharide largely associated with invertebrate exoskeletons. Thus, chitinous organisms with greater surface area to volume ratio should have higher amounts of both elements. For example, the families with the highest %N were represented by shrimps (e.g., Stenopodidae and Penaeidae, mean %N = 11.1 and 10.4, %C = 46.6 and 37.4, respectively) and lobsters (Palinuridae, mean %N = 12.9, %C = 43.2) with high surface area to volume ratio and a chitinous shell. In contrast, the jellyfish family Cassiopeidae, with no chitinous material, had among the lowest %C = 14.9 and %N = 3.07. We also found only weak support for our hypothesis that trophic group would explain variation in the slopes of these relationships, providing further support that dietary restrictions do not appear to be key drivers of the composition of body nutrients across a diverse group of organisms. Instead, the finding that taxonomic identity (at the family level) was a superior predictor highlights the key take-home message from our study: taxonomic identity is the baseline from which to predict stoichiometric relationships among elements.

## Supplementary information

Supplementary file1 (XLS 197 kb)

## Data Availability

The data supporting the results is available as supplementary information to this paper.
